# A Topographical Anatomical Description of the Orbit of the Alpaca, *Vicugna pacos*

**DOI:** 10.1111/vop.70270

**Published:** 2026-09-18

**Authors:** Sophia Weis, Johann Maierl

**Affiliations:** ^1^ Institute of Veterinary Anatomy, University of Munich (LMU) Munich Germany

**Keywords:** musculi bulbi, nervi craniales, ocular region, *orbita*, South American camelids, veterinary anatomy

## Abstract

**Objective:**

To describe the gross anatomy of the *orbita* in Huacaya alpacas, identify species‐specific features, and provide comparative anatomical information relevant to clinical ophthalmic practice.

**Animals Studied:**

Nine heads from postmortem donated Huacaya alpacas were used.

**Procedures:**

Two skulls were evaluated to characterize the *orbita*, and six additional heads were analyzed using gross dissection and saw sectioning.

**Results:**

The *orbita* is completely enclosed by bone and protects the *mm. bulbi* and associated *nn. craniales*. The *mm. bulbi* originate mainly around the *can. opticus* and *for. orbitorotundum*, except for the *m*. *obliquus ventralis,* that arises from the *os lacrimale*. Orbital nerves include the *n*. *opticus*, the ocular motor nerves (*n. oculomotorius*, *n. trochlearis*, *n. abducens*), as well as the *n. ophthalmicus* and *n. maxillaris*. With the exception of the *m. obliquus dorsalis*, *m. rectus lateralis,* and the lateral part of the *m. retractor bulbi*, all *mm. bulbi* are innervated by the *n. oculomotorius*. These exceptions are innervated by the *n. trochlearis* and *n. abducens*, respectively.

**Conclusions:**

The macroscopic anatomy of the *orbit*a in Huacaya alpacas largely corresponds to that of domestic animals. However, novel details were identified, including bifurcation of the *n. trochlearis* near the *m. obliquus dorsalis* and fine connecting branches of the *n. ophthalmicus* to adjacent structures. These features warrant further study to support veterinary care and improve understanding of alpaca orbital anatomy.

## 1 Introduction

The evolution of the Camelidae began approximately 40 million years ago in North America, with numerous genera either migrating or becoming extinct. Those that migrated from North America to South America evolved around 2 million years ago into what are now known as the South American camelids (SACs) [1].

Alpacas ( *Vicugna pacos* )—as part of the SACs—were first exported from South America in the 19th century [2]. Over time, interest in alpacas in Germany has increased as a result of both commercial and private husbandry [3].

Alpacas are characterized by the appearance of their eyes, that seem disproportionately large in relation to their comparatively small heads. However, their relative eye size is comparable to that of horses and cattle [4].

Nevertheless, ophthalmologic injuries occur relatively frequently in alpacas [4, 5]. These include congenital malformations such as entropion, ectropion, and eyelid cysts, as well as infectious or traumatic conditions. Displacement of the *ductus nasolacrimalis* is also frequently observed and may lead to chronic lacrimation and recurrent inflammation. Furthermore, conjunctivitis caused by bacterial infection or trauma is common. When alpacas are kept together with equids, EHV‐1‐associated diseases of the posterior segment of the eye may also occur [4]. In general, ocular diseases may impair visual function and adversely affect overall wellbeing while also posing substantial economic and management challenges for the owner [6].

Adequate medical management of disorders affecting the orbital region requires thorough anatomical knowledge. In clinical practice, a precise topographical anatomical description of the *orbita* is essential to minimize the risk of iatrogenic injury and to ensure accurate surgical orientation in this anatomically complex region. In particular, macroscopic examination of the *musculi* (*mm.) bulbi* and their innervation represents an essential basis for surgical interventions in this area [7].

In addition, detailed knowledge of anatomical landmarks is crucial for the administration of local anesthesia. A local anesthetic technique used in ruminants for ocular surgery is the Peterson nerve block [8, 9]. This nerve block is performed by depositing the anesthetic just anterior to the *foramen* (*for.) orbitorotundum* to block all nerves exiting this foramen. By inserting a curved needle just posterior to the junction of the *arcus zygomaticus* and the *processus (proc.) zygomaticus ossis frontalis*, the needle can be advanced toward the *proc. coronoideus mandibulae*. From there, the needle is guided along the anterior border of the latter to reach the bony plate that forms the *fossa pterygopalatina* [9]. A detailed, species‐specific anatomical description is therefore crucial for the safe and effective application of regional anesthetic techniques.

With sufficient knowledge, this nerve block may also be applied to the alpaca when indicated. To date, however, the literature lacks a comprehensive topographical anatomical description of the alpaca *orbita*. Although the literature on alpaca anatomy remains sparse overall, the body regions that have been investigated, such as the vertebral column, the stomach, and limbs, have revealed several species‐specific morphological features [10, 11, 12]. Given the prevalence of anatomical variation among mammals, it remains unclear whether the anatomy of the alpaca *orbita* differs from that described in other domestic species, highlighting a significant knowledge gap regarding this functionally important anatomical region.

This study aims to address this gap by describing the topographical anatomy of this region and comparing it with previously described findings in other mammals.

## 2 Material and Methods

A total of nine Huacaya alpaca heads were examined. The analyzed specimens consisted of two skulls and seven heads. All specimens were obtained through postmortem donations from animals ranging in age from 6 months to 16 years. These were examined by means of gross dissection and saw cut techniques. Table 1 summarizes the heads utilized in this study and the corresponding investigations conducted for each. The two skulls are not included, as they did not require any additional investigative methods.

**TABLE 1:** Huacaya Alpaca heads used for gross dissection and the methods applied.

Race	Age (year)	Gender	Investigation
Huacaya	0.5	Female	Arterial latex milk injection, *orbita* opened dorsally
Huacaya	2.5	Female	*Orbita* opened dorsally
Huacaya	10	Male neutered	*Orbita* opened dorsally
Huacaya	2	Unknown	Bisected longitudinally along the median plane
Huacaya	4	Male neutered	Arterial latex milk injection, bisected longitudinally along the median plane
Huacaya	13	Male	Horizontal saw cuts
Huacaya	15	Male neutered	Arterial latex milk injection, saw cuts: left half horizontally, right half transversally

### 2.1 For Animal Procurement

No animals were euthanized for the purpose of this study. Only postmortem carcass donations from private owners or veterinary clinics were used. Therefore, approval from an ethics committee was not required. The animals included in this study had either died of natural causes or had been euthanized by their attending veterinarians for reasons unrelated to the region under investigation. Subsequently, the specimens were transported to the Department of Veterinary Anatomy for preparation and dissection.

### 2.2 Storage

Specimens not immediately utilized were preserved by freezing at −20°C. Specimens under processing were stored in 40 L of 10% brine solution, comprising water and nitrite curing salt („Südwestdeutsche Salzwerke AG Nitrit Pökelsalz jodiert 0,5%–0,6%“), maintained within a cooling chamber at 4°C. To minimize oxygen exposure, the brine surface was tightly covered with a plastic film.

### 2.3 Preparation

Oscillating saws („Elektronik Power Autopsiesäge Art.‐Nr. 04.20.00, 230 V, 50 Hz, 500 W“, with attachment: „MST‐Instrumente GmbH, REF 02.00.30 Sägeblatt Segment 50/65 mm, unbeschichtet“) and milling cutters („PROXXON S.A. Netzgerät AC/DC NO 28505‐11, 230 V, 50/60 Hz“, with attachment: „PROXXON NO 28 234 Diamant Schleifstifte Ø 3,2 mm; PROXXON NO 29 062 Raspelfräser mit Metallnadeln aus Wolfram‐Karbid 8 × 12 mm; Donau Elektronik GmbH NO 1641 Kreissägeblätter Ø 16mm, Ø 22 mm“) were used for specimen preparation, followed by standard dissecting tools.

In three specimens, the heads were opened dorsally. For this purpose, the skull, brain, *m. temporalis*, and the *proc. coronoideus mandibulae* were removed. In two specimens, the heads were bisected longitudinally along the median plane. In these, the (hemi‐)brain was removed from the medial aspect, while the *m. temporalis* and the *proc. coronoideus mandibulae* were removed from the lateral aspect. In addition, the *os frontale* and *arcus zygomaticus* were milled away.

To enhance visualization of muscular insertions during macroscopic preparation, the collapsed *bulbi oculi* of all macroscopically prepared heads were injected with approximately 3 mL of 10% brine solution. To harden the *nn. craniales* that had become markedly softened as a result of postmortem decomposition, approximately 2 mL of a 10% brine solution containing 7% formalin were injected into the nerves using a syringe.

### 2.4 Latex Injection

For improved visualization of the arterial vessels, approximately 35 mL of red latex milk („Laguna Naturlatex; Colok GmbH Naturlatex“) was bilaterally injected into the *arteria (a.) carotis communis* of three heads using disposable syringes and blunt‐tipped cannulas. Following complete hardening of the latex milk, which required approximately 48 h in the cold room, the specimens were rendered suitable for subsequent examination.

### 2.5 Saw Cuts

For the production of saw cuts, two heads were immersed in water and frozen to ensure homogeneous freezing throughout the specimen. The even distribution of water over the external surfaces and within the internal cavities of the head provided uniform support to both soft and mineralized tissues during freezing. This facilitated the production of smooth, well‐preserved saw sections while minimizing tissue disruption and preventing the displacement of fragmentation of anatomical structures. The heads, embedded within ice blocks, were sectioned into slices approximately 1 cm thick using a band saw equipped with a spacer („C.‐E. Reich GmbH, Bandsäge 4210“). The individual slices were maintained in a frozen state until photographed. To rinse impurities from the surface of a slice prior to photography, 50% ethanol was applied using a wash bottle. The procedure was then repeated on the opposite side. All slices from both heads were documented photographically in this manner.

### 2.6 Photo Documentation

A digital camera („Olympus Digital Camera Modell E‐620 mit Olympus Digital ED 14‐42mm 1:3.5‐5.6 Objektiv“) was used to document the specimens. To reduce highlights, polarizing filters („hama PL CIR Ø 58mm; Heliopan Polarisationsfilterfolien FS‐Nr. 8006 0,4mm Dicke“) were used. The specimens were photographed against a black background and subsequently post‐processed with Adobe Photoshop Version 25.5.1.

## 3 Results

### 3.1 Bony Boundary of the *Orbita*

The *orbita* of the alpaca is formed by bones of the *ossa faciei* and the *ossa cranii*. The bones contributing to its structure include the *os lacrimale*, *os zygomaticum*, *maxilla*, *os palatinum*, *os frontale*, *os temporale*, and *os sphenoidale*.

**FIGURE 1:**
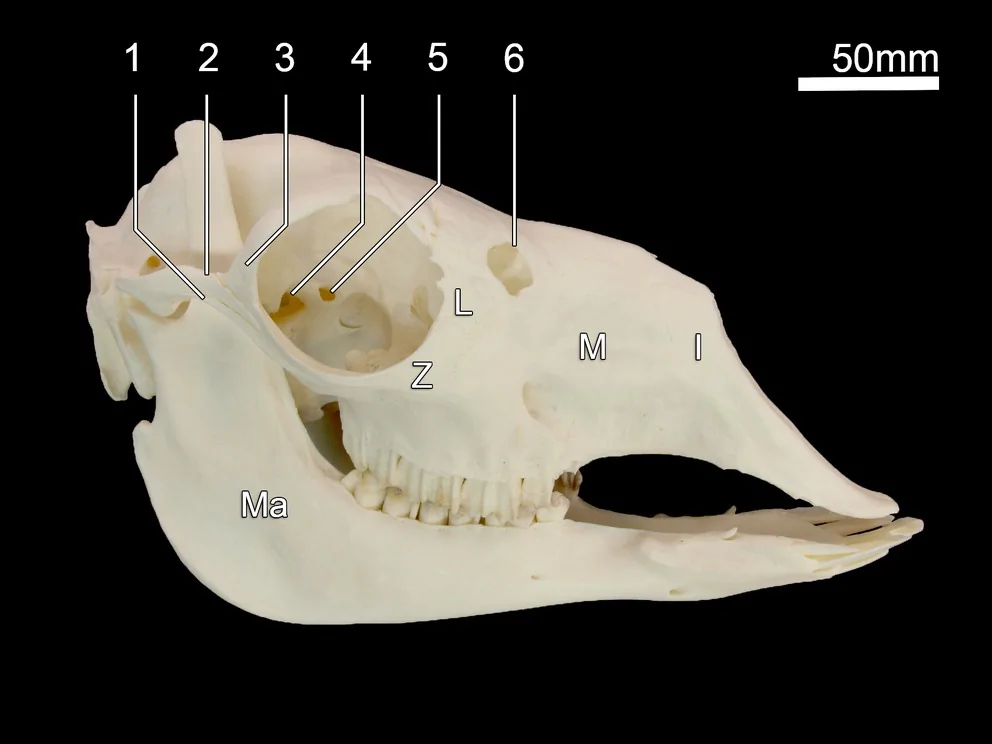
Skull of an Alpaca (lateral view from the right side) 1, Processus temporalis ossis zygomatici; 2, Processus zygomaticus ossis temporalis; 3, Processus zygomaticus ossis frontalis; 4, Foramen orbitorotundum; 5, Canalis opticus; 6, “Fontanela lagrimal”; I, Os incisivum; L, Os lacrimale; M, Maxilla; Ma, Mandibula; Z, Os zygomaticum.

The dorsal part of the *orbita* is formed by the *os frontale*. Its *proc. zygomaticus* (Figure 1) connects with the *proc. frontalis* of the *os zygomaticum*, thereby closing the bony *aditus orbitae*. Dorsomedially at the base of the *proc. zygomaticus* lies the *fovea trochlearis* for the *trochlea* of the *musculus* (*m.) obliquus dorsalis*. The *for. supraorbitale* is located dorsally and communicates with the *orbita* via the *canalis* (*can.) supraorbitalis*. The *sulcus supraorbitalis* runs rostrally from the *foramen*. The *incisura infratrochlearis*, located dorsal to the *os lacrimale*, is also found within the region of the *os frontale*.

**FIGURE 2:**
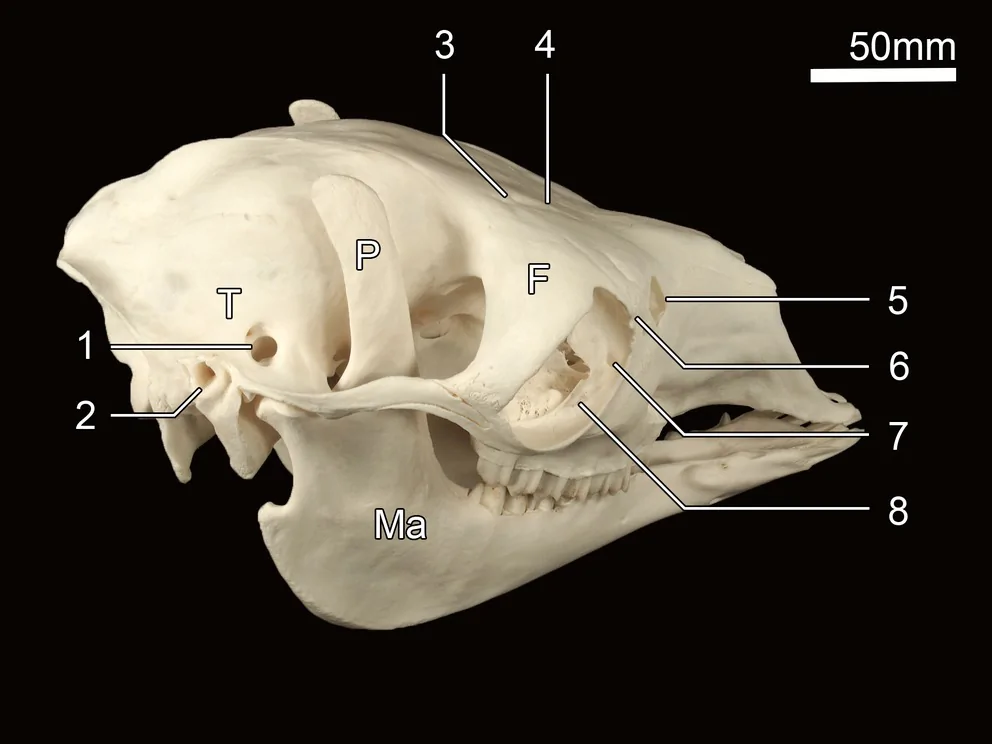
Skull of an Alpaca (dorsolateral view from the right side) 1, Foramen temporale; 2, Meatus acusticus externus; 3, Foramen supraorbitale; 4, Sulcus supraorbitalis; 5, “Fontanela lagrimal”; 6, Incisura infratrochlearis; 7, Fossa sacci lacrimalis; 8, Fossa musculi obliqui ventralis; F, Processus zygomaticus ossis frontalis; Ma, Mandibula; P, Processus coronoideus mandibulae; T, Os temporale.

Rostrally, the *os lacrimale* (Figure 1) follows. Its *facies orbitalis* contains the *fossa sacci lacrimalis*, providing access to the *ductus nasolacrimalis*, as well as the *fossa musculi obliqui ventralis*, serving as the site of origin for the *m. obliquus ventralis*. Rostrodorsal to the *os lacrimale*, an opening (Figure 2) is identifiable, allowing direct access for probing the *sinus frontalis* on the macerated skull. This opening has previously been described as “fontanela lagrimal” (Term not recognized in the NAV) [13, 14, 15].

The *os zygomaticum* contributes to the boundary of the *aditus orbitae* via its *proc. frontalis* and participates in the *arcus zygomaticus* through the *proc. temporalis* (Figure 1). The *proc. zygomaticus* of the *os temporale* (Figure 1) does not extend to the *margo orbitalis* in the alpaca and is therefore not involved in forming the boundary of the *aditus orbitae*.

The *margo orbitalis* forms a complete bony ring, separating the *facies orbitalis* from the *facies facialis*. It is subdivided into the *margo supraorbitalis* and the *margo infraorbitalis*.

The medial wall of the *orbita* is formed by the *facies orbitalis* of the *os frontale* and *os lacrimale*, the *ala ossis praesphenoidalis*, and the *lamina perpendicularis* of the *os palatinum*. The ventral wall is constituted by the *facies orbitalis* of the *maxilla*.

**FIGURE 3:**
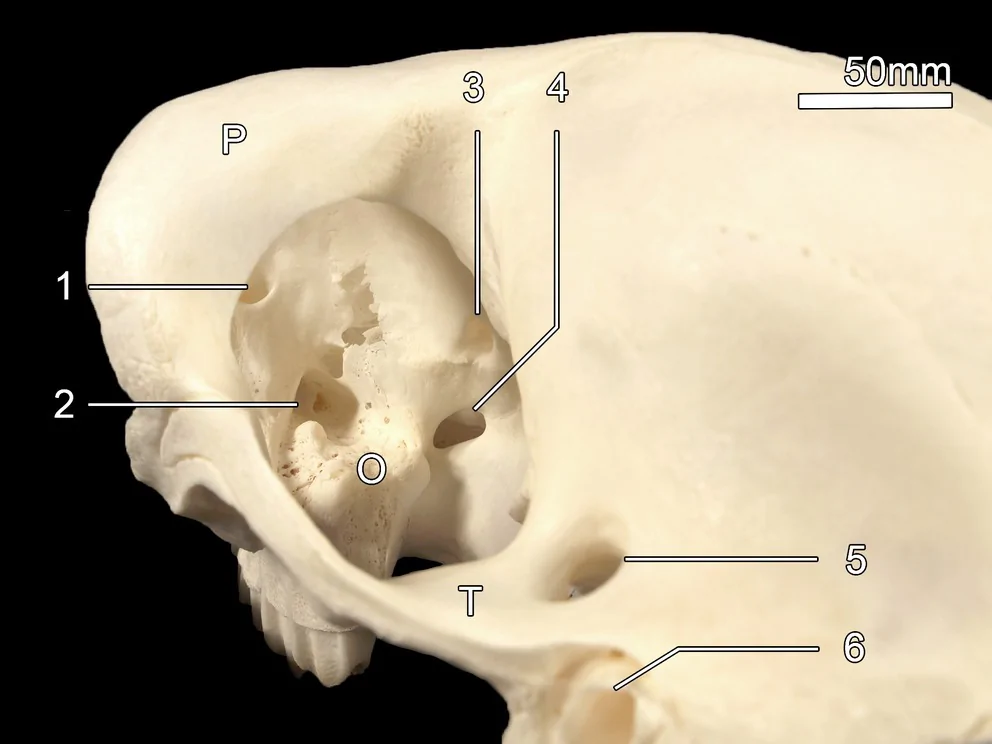
Skull of an Alpaca, lower jaw removed (caudolateral view from the left side): 1, Fossa sacci lacrimalis; 2, Foramen maxillare; 3, Foramen ethmoidale; 4, Foramen sphenopalatinum; 5, Foramen temporale; 6, Meatus acusticus externus; O, Facies orbitalis maxillae; P, Processus zygomaticus ossis frontalis; T, Os temporale.

Several openings are present in the medial wall: The *for. ethmoidale* (Figure 3) is located between the *os frontale* and *os praesphenoidale*. The *can. opticus* (Figure 1) is located within the *os praesphenoidale*, allowing the *nervus* (*n.) opticus* and *a. ophthalmica interna* to pass. The *for. orbitorotundum* (Figure 1) is found at the junction of the *os basisphenoidale* and *os praesphenoidale*, allowing passage for the *n. oculomotorius*, *n. trochlearis*, *n. abducens*, *n. ophthalmicus*, and *n. maxillaris*. The *for. maxillare* (Figure 3) on the *facies orbitalis* of the *maxilla* transmits the *n. maxillaris* and accompanying vessels rostrally.

Dorsal to this lies the longitudinal, oval *for. sphenopalatinum* (Figure 3), providing direct communication with the *cavum nasi*. This *foramen* is bordered by the *os lacrimale*, *os frontale*, *lamina perpendicularis* of the *os palatinum*, and the *maxilla*.

The *for. palatinum caudale* is located on the *lamina perpendicularis* of the *os palatinum*.

### 3.2 About the *Mm. bulbi*

The *mm. bulbi* of the alpaca consist of the four *mm. recti* (*lateralis*, *dorsalis*, *ventralis*, *medialis*), the two *mm. obliqui* (*dorsalis* and *ventralis*), the *m. retractor bulbi*, and the *m. levator palpebrae superioris*. These muscles enable motility of the *bulbus oculi* and elevation of the upper eyelid. They are enclosed by the firm *periorbita* and separated from one another by adipose tissue, as part of the *corpus adiposum orbitae* and delicate fascia.

**FIGURE 4:**
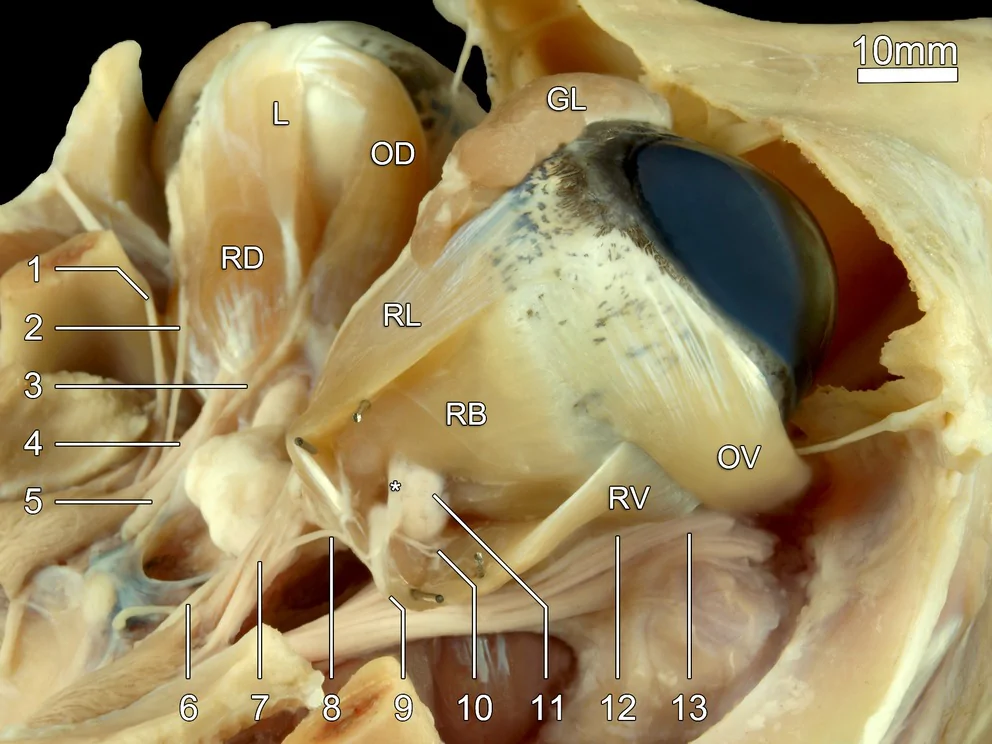
Caudolateral view from the right side of an alpaca head following removal of the skullcap and brain, right eye muscle pyramid rotated anti‐clockwise around the longitudinal axis 1–5, left; 1, Nervus zygomaticus; 2, Nervus lacrimalis; 3, Nervus trochlearis; 4, Nervus ophthalmicus; 5, Nervus oculomotorius; 6–13, right; 6, Nervus oculomotorius; 7; 8, Nervus ophthalmicus; 7, Nervus ophthalmicus; 8, Branch to the ramus ventralis nervi oculomotorii; 9, Ramus ventralis nervi oculomotorii on the musculus rectus ventralis; 10, Innervation of the musculus rectus ventralis by branches of the ramus ventralis nervi oculomotorii; 11, Nervus opticus; 12, Terminal branch of the ramus ventralis nervi oculomotorii on its way to the musculus obliquus ventralis; 13, Nervus maxillaris; *, Branch of the ramus ventralis nervi oculomotorii to the musculus retractor bulbi, GL, Glandula lacrimalis; L, Musculus levator palpebrae superioris; OD, Musculus obliquus dorsalis; OV, Musculus obliquus ventralis; RB, Musculus retractor bulbi; RD, Musculus rectus dorsalis; RL, Musculus rectus lateralis; RV, Musculus rectus ventralis.

**FIGURE 5:**
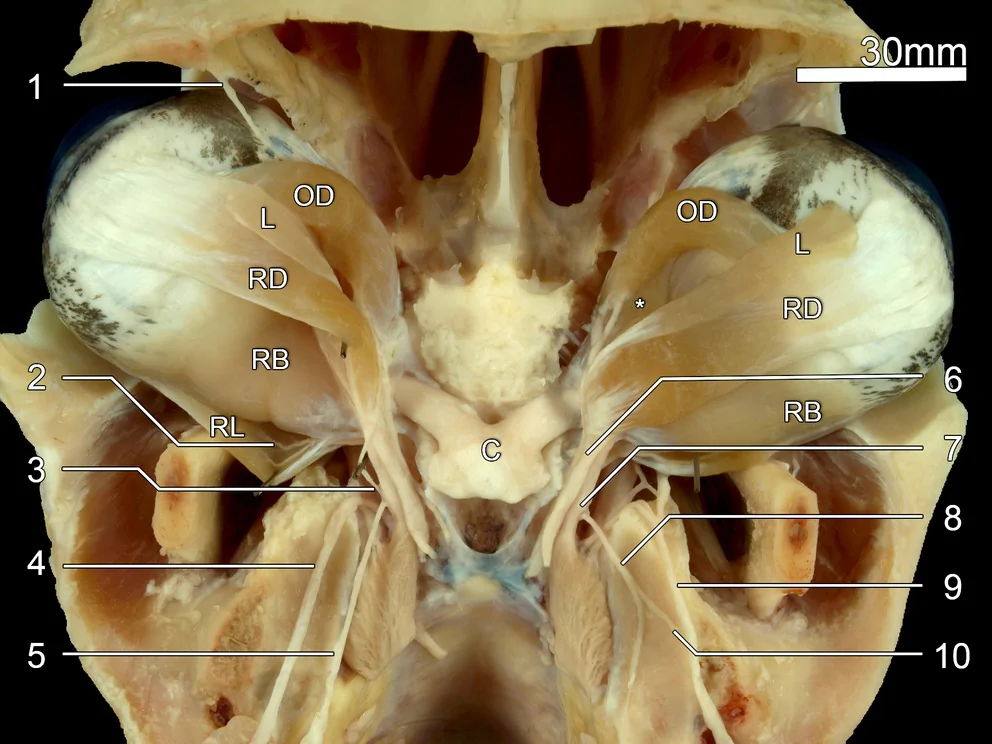
Dorsal view of an alpaca head after removal of the skullcap, brain and glandulae lacrimales 1–5, left; 1, Nervus frontalis; 2, Innervation of the musculus rectus lateralis by the nervus abducens; 3, Branch of the nervus ophthalmicus to the musculus rectus dorsalis; 4, Nervus zygomaticus; reflected caudally; 5, Nervus lacrimalis; reflected caudally; 6–10, right; 6, Nervus oculomotorius; 7, Nervus ophthalmicus; 8, Nervus lacrimalis; reflected caudally; 9, Nervus zygomaticus; reflected caudally; 10, Ramus communicans cum nervi lacrimali of the nervus zygomaticus; *, Musculus rectus medialis; C, Chiasma opticum; L, Musculus levator palpebrae superioris; OD, Musculus obliquus dorsalis; RB, Musculus retractor bulbi; RD, Musculus rectus dorsalis; RL, Musculus rectus lateralis.

**FIGURE 6:**
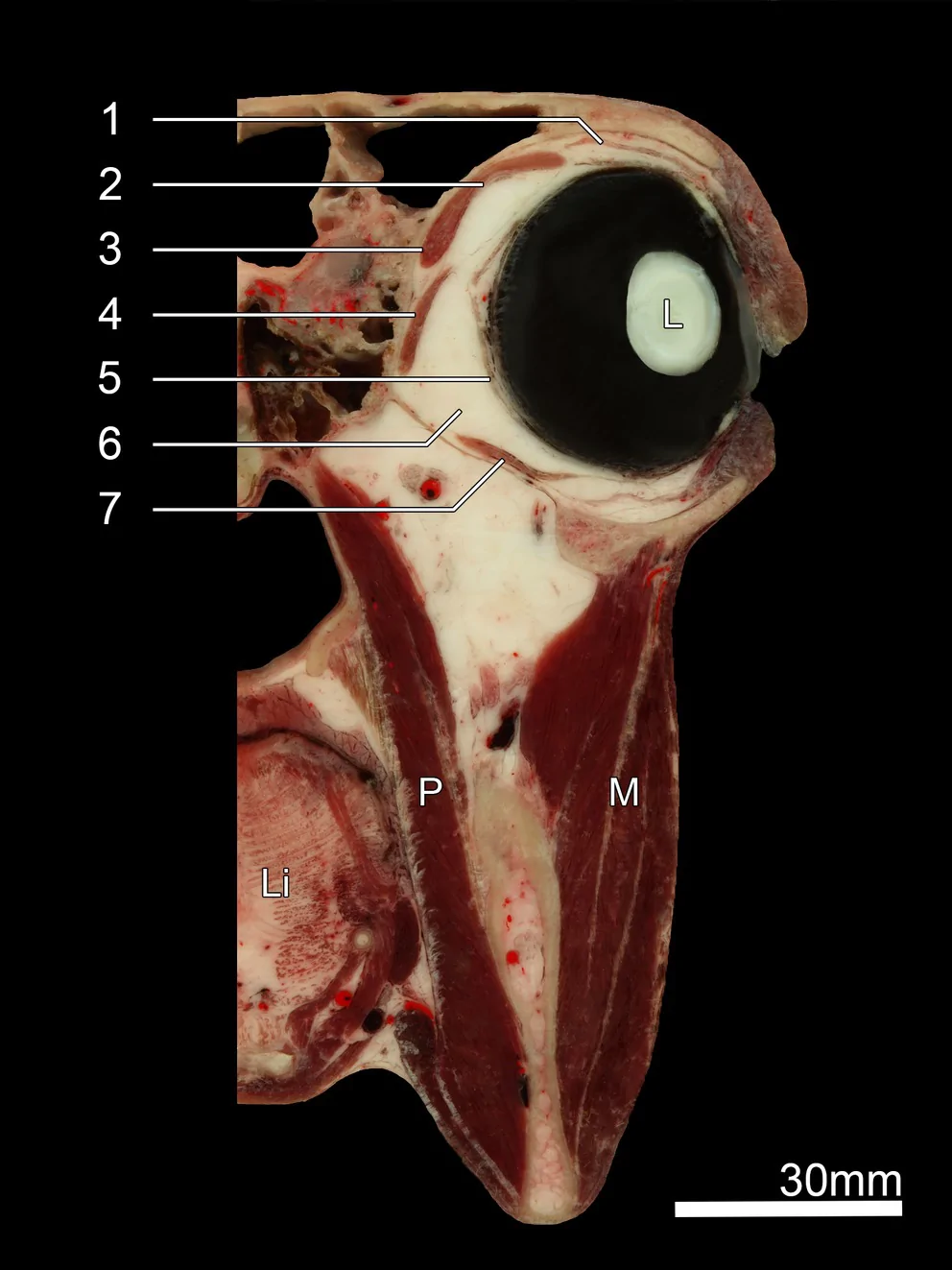
Transverse section of half an alpaca head (rostral view of the left half): 1, Musculus levator palpebrae superioris; 2, Trochlea; 3, Musculus obliquus dorsalis; 4, Musculus rectus dorsalis; 5, Musculus retractor bulbi; 6, Corpus adiposum orbitae; 7, Musculus rectus ventralis; L, Lens; Li, Lingua; M, Musculus masseter; P, Musculus pterygoideus medialis.

The *mm. recti* (Figures 4 and 5) originate collectively in a fibrous ring located rostrolateral to the *can. opticus* and rostral to rostromedial to the *for. orbitorotundum*. As indicated by their names, these muscles course in their respective directions and insert on the *sclera*. The *m. levator palpebrae superioris* (Figures 5 and 6) arises from the same region, lies dorsomedially to the *m. rectus dorsalis*, and radiates into the *palpebra superior*.

**FIGURE 7:**
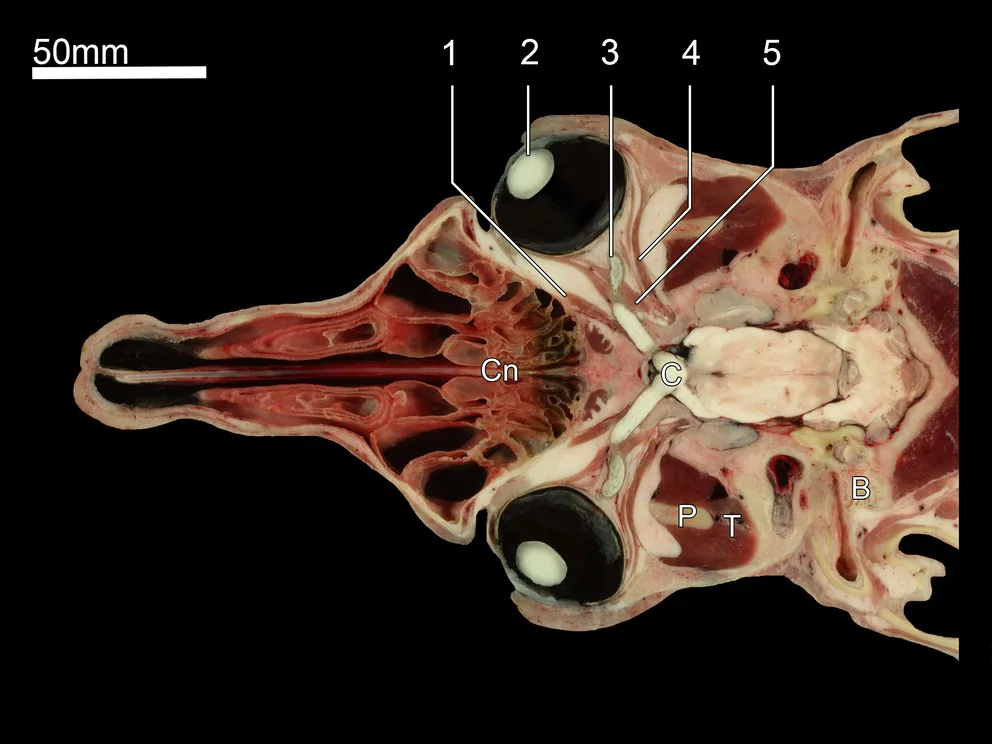
Horizontal section of an alpaca head (dorsal view): 1, Musculus rectus medialis (right); 2, Lens; 3, Nervus opticus; 4, Musculus rectus lateralis; 5, Musculus retractor bulbi; B, Bulla tympanica; C, Chiasma opticum; Cn, Cavum nasi; P, Processus coronoideus mandibulae; T, Musculus temporalis.

The *m. retractor bulbi* (Figures 4 and 7) is located beneath the *mm. recti* and a substantial layer of adipose tissue. Upon removal of these muscles and the fat tissue, a uniform muscle becomes apparent, with its fiber bundles arranged in a conical shape. The *n. opticus* passes through a slit‐shaped aperture within the muscle and is subsequently encircled by the *m. retractor bulbi*. The muscle inserts caudally on the *sclera*.

The *m. obliquus dorsalis* (Figures 4 and 6) also originates in the region of the *for. orbitorotundum* and courses rostrodorsally along the medial wall of the *orbita*. It is deflected laterally by the cartilaginous *trochlea* (Figure 6) and passes beneath the *m. rectus dorsalis* lateral to the *sclera*.

The *m. obliquus ventralis* (Figure 4) arises from the *fossa musculi obliqui ventralis* of the *os lacrimale* and runs ventrolaterally over the insertion of the *m. rectus ventralis* to attach to the *sclera* at the level of the *m. rectus lateralis*.

### 3.3 Regarding the *Nn. craniales* within the *Orbita*

The *n. opticus* (II) (Figures 4 and 7) lies medial to the *n. oculomotorius*. It is enveloped by a thin connective tissue sheath in both the intraorbital and intracranial segments. Following removal of the bony *can. opticus* and the *vaginae nervi optici*, the precise course of the nerve can be more clearly described.

After exiting the cranial cavity via the *can. opticus*, the *n. opticus* proceeds caudally to the region of origin of most of the *mm. bulbi* and passes beneath the *m. obliquus dorsalis*, *m. rectus dorsalis*, and *m. levator palpebrae superioris*. It is subsequently enveloped by these muscles and fat tissue before entering the muscular cone of the *m. retractor bulbi* caudomedially, where it is encircled by its fiber bundles. The *n. opticus* then continues caudoventrally toward the *bulbus oculi*, entering it in the ventrolateral quadrant.

**FIGURE 8:**
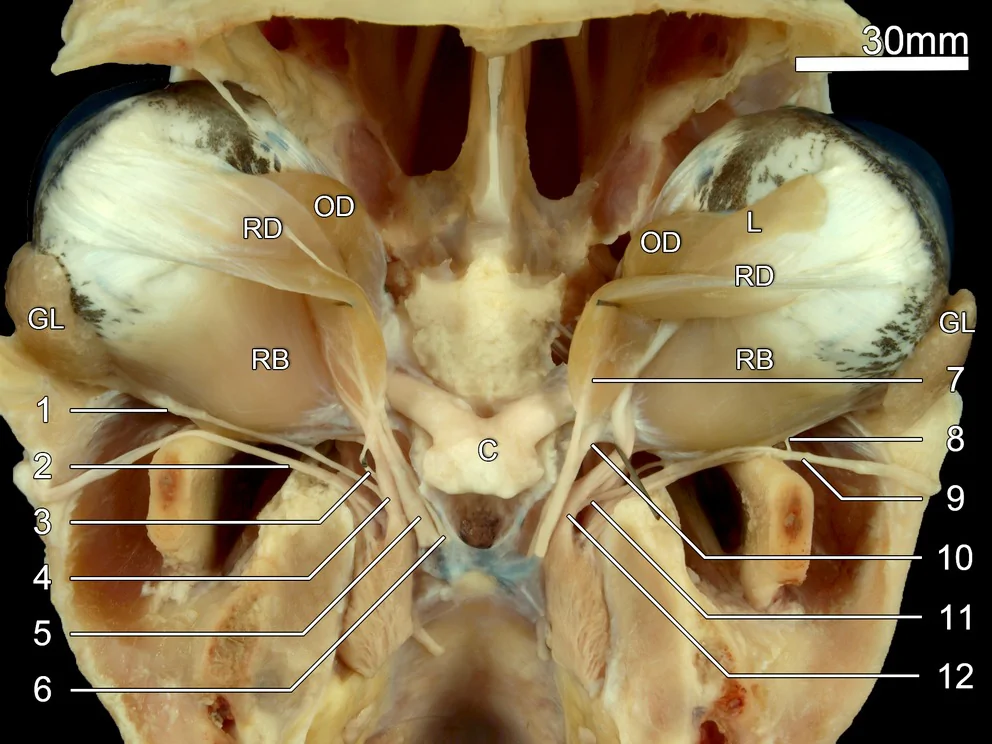
Dorsal view of an alpaca head following removal of the skullcap and brain 1–6, left; 1, Nervus lacrimalis; 2, Nervus zygomaticus; 3, Branch of the nervus ophthalmicus to the musculus rectus dorsalis; 4, Nervus ophthalmicus; 5, Nervus oculomotorius; 6, Nervus trochlearis; 7–12, right; 7, Innervation of the musculus rectus dorsalis by the ramus dorsalis nervi oculomotorii; 8, Ramus communicans cum nervi lacrimali of the nervus zygomaticus; 9, Nervus zygomaticus; 10, Ramus ventralis nervi oculomotorii; 11, Nervus lacrimalis; 12, Nervus ophthalmicus; C, Chiasma opticum; GL, Glandula lacrimalis; L, Musculus levator palpebrae superioris; OD, Musculus obliquus dorsalis; RB, Musculus retractor bulbi; RD, Musculus rectus dorsalis.

The *n. oculomotorius* (III) (Figure 8) passes through the *for. orbitorotundum* together with the *n. trochlearis* (IV), *n. abducens* (VI), and two branches of the *n. trigeminus*—the *n. ophthalmicus* (V_1_) and *n. maxillaris* (V_2_).

Upon exiting this opening, the *n. oculomotorius* courses rostrally, medial to the branches of the *n. trigeminus*, and shortly thereafter divides into a slender *ramus* (*r.) dorsalis* (Figure 8) and a stronger *r. ventralis* (Figure 8).

The *r. dorsalis* crosses superficial to the *n. ophthalmicus* and extends caudally into the medial aspect of the *m. rectus dorsalis*, adjacent to the *m. retractor bulbi*. Here, it branches into terminal fibers, some of which penetrate the *m. rectus dorsalis* to innervate the *m. levator palpebrae superioris*.

The *r. ventralis* courses rostrally, medial to the *n. ophthalmicus*, into the region of origin of the *mm. bulbi*, enters the *m. retractor bulbi*, similarly to the *n. opticus*, and crosses the *n. opticus*. Caudolateral to the *n. opticus*, it reaches the ventral portion of the *m. retractor bulbi*, where it divides into branches supplying the *m. rectus medialis*, *m. rectus ventralis* (Figure 4), and *m. retractor bulbi* (Figure 4). The branch to the *m. retractor bulbi* is particularly delicate and divides into fine filaments that cannot be further followed macroscopically.

The terminal branch of the *r. ventralis* runs along the caudal border of the *m. rectus ventralis*, crosses it rostroventrally (Figure 4), lies upon it, and continues (Figure 4) into the caudal border of the *m. obliquus ventralis*. As this terminal branch crosses to the external surface of the *m. rectus ventralis*, a fine branch of the *n. ophthalmicus* becomes visible, dividing into two filaments before reaching the muscle (Figure 4). One filament appears to approach the terminal branch of the *r. ventralis*, continuing to the *m. rectus ventralis*. The other filament proceeds toward the *m. retractor bulbi*. The course of the latter branches could not be further traced.

The *n. trochlearis* (IV) (Figures 4 and 8) courses rostrally over the *n. trigeminus*, crosses the *n. oculomotorius* and the region of origin of the *mm. bulbi* in a dorsolateral direction. It then extends caudally into the *m. obliquus dorsalis*. In two specimens, a bifurcation was observed before the nerve entered the muscle; in three specimens, it remained externally uniform but divided into two branches within the muscle belly.

Although the *n. trigeminus* (V) is not an ocular motor nerve, it is prominently represented within the *orbita*.

The *n. ophthalmicus* (V_1_) (Figures 4 and 8) gives off a fine branch that courses laterally to the *m. rectus dorsalis* and extends into the *glandula (gl.) lacrimalis* at its lateral margin. This branch corresponds to the *n. lacrimalis* (Figures 4 and 8). The *gl. lacrimalis* is situated dorsolaterally on the *mm. bulbi* (Figures 4 and 8). The *n. ophthalmicus* continues between the two branches of the *n. oculomotorius* and passes beneath the caudal border of the *m. rectus dorsalis*.

Before it passes between the two branches, the *n. ophthalmicus* gives off a fine branch that crosses over the *r. dorsalis* of the *n. oculomotorius* and inserts at the caudal edge of the *m. rectus dorsalis* (Figures 5 and 8). Ultimately, the *n. ophthalmicus* divides below the *m. rectus dorsalis* and *m. obliquus dorsalis* into the *n. frontalis* (Figure 5) and the *n. nasociliaris*. The *n. nasociliaris* could not be visualized in the images obtained.

The *n. frontalis* proceeds rostrodorsally, runs over the *m. rectus medialis* and exits the *orbita* at the dorsal bony margin. The *n. nasociliaris* runs medial to the *m. obliquus dorsalis*, subsequently dividing into the *n. ethmoidalis* that passes through the *lamina cribrosa* and the *n. infratrochlearis*, proceeding to the medial corner of the eye.

The *n. maxillaris* (V_2_) (Figure 4) courses rostrally, lateral to the *n. ophthalmicus*, passes beneath the *mm. bulbi*, and continues rostrally, covered by fat tissue. The *n. zygomaticus* (Figures 4 and 8) arises from the *n. maxillaris* and, accompanied by blood vessels, pierces the *periorbita* laterally. It gives off a fine *r. communicans cum n. lacrimali* (Figures 5 and 8) to the *n. lacrimalis*. The *r. zygomaticotemporalis* curves around the *arcus zygomaticus*, whereas the *r. zygomaticofacialis* lies beneath the *palpebra inferior*.

Further branches of the *n. maxillaris* include the *n. pterygopalatinus* (with the *n. nasalis caudalis*) and the *n. infraorbitalis*, that represents the rostral continuation of the *n. maxillaris*.

The *n. abducens* (VI) (Figure 5) is positioned ventromedial to the *n. trigeminus*, concealed beneath arterial vessels. It courses rostrally, penetrates the tendinous origin of the *m. rectus lateralis* at its dorsal margin, and divides into two to three fine branches: one to two of these branches supply the medial aspect of the *m. rectus lateralis*, and one delicate branch is directed to the lateral portion of the *m. retractor bulbi*.

## 4 Discussion

Anatomical research offers a macroscopic description of the very inner workings—the machinery—of life.

Applications go beyond satisfying our scientific curiosity: Anatomy lays the theoretical foundation for a vast number of clinical procedures and practical treatments. Anatomical variations between and within species may substantially influence diagnostic interpretation, surgical access, regional anesthesia, and the clinical management of pathological conditions. Moreover, direct extrapolation from other domestic species may be inappropriate in the presence of distinct morphological adaptations. However, to date, research on the anatomical particularities of the alpaca remains sparse. This is especially true for special regions like the *orbita*. Such gaps in knowledge can cause complications when deviations are overlooked and therefore undermine confidence in clinical decision‐making.

Our findings indicate that the alpaca exhibits only minor variation in the osseous, muscular, and neural structures of the *orbita* compared with other domestic animals. Nevertheless, several subtle but noteworthy differences were identified. The close overall correspondence suggests that many diagnostic and therapeutic approaches established for other domestic species may also be applicable to alpacas. However, the species‐specific anatomical characteristics identified in the present study should be taken into account when adapting these techniques, particularly with regard to advanced imaging interpretation, ophthalmic and orbital surgical approaches, and regional anesthesia. A comprehensive understanding of normal anatomy also enhances the interpretation of cross sectional imaging, such as CT and MRI, by providing a reliable reference for distinguishing normal anatomical structures from pathological alterations.

Detailed anatomical knowledge is fundamental to the safe and effective performance of clinical procedures involving the *orbita*. In cattle, for instance, regional anesthetic techniques such as the Peterson nerve block and retrobulbar block are routinely employed for ophthalmic procedures [8]. However, even in this well‐studied species, complications including inadvertent injection into adjacent structures, orbital hemorrhage, globe penetration, and optic nerve injury have been reported [8], highlighting the critical importance of precise anatomical knowledge. Likewise, a thorough understanding of the anatomy of the *orbita* is essential for surgical planning, interpretation of diagnostic imaging, and minimizing the risk of iatrogenic injury during ophthalmic interventions.

The anatomical data presented in this study provide a species‐specific reference for the alpaca *orbita* and address the current lack of detailed anatomical information available for this increasingly important domestic species. By establishing a comprehensive anatomical baseline, this study may facilitate future clinical, diagnostic, and surgical applications while providing a foundation for further research in alpaca ophthalmology.

The following section details both the similarities and distinctive anatomical features observed in the alpaca *orbita* in comparison with other domestic mammals.

### 4.1 To the Bony *Orbita*

When comparing animal species, different configurations of the bony orbita can be observed. In camelids, the orbita is completely enclosed by bone, providing additional ocular protection through prominent supraorbital ridges [5]. Horses and ruminants also exhibit a fully ossified aditus orbitae, making them comparable [16, 17].

In the alpaca, the *for. supraorbitale*, located dorsally on the *os frontale*, corresponds to that of the horse, where it is positioned at the origin of the *proc. zygomaticus ossis frontalis*. This opening is absent in carnivores, whereas in ruminants it is situated medially on the roof of the skull [17]. The rostrally extending *sulcus supraorbitalis* is also present in pigs and small ruminants, in addition to alpacas [17]. The *for. supraorbitale* and the rostrally extending *sulcus supraorbitalis* of the alpaca are also described in the lama [18].

The opening on the skull found dorsal to the *os lacrimale* was already described in a previous study on the alpaca as the “fontanela lagrimal” [19]. The llama also presents a similar opening. It has been described as the “fronto‐naso‐lacrimo‐maxillary foramen,” and also appears in deer, Bactrian camels, and dromedaries [18]. This area may serve to harbor preorbital glands, such as those found in axis deer [20] or Bardoka sheep [21].

Unlike the horse, in the alpaca, the *proc. zygomaticus ossis temporalis* does not reach the *margo orbitalis* and therefore does not contribute to the formation of the *aditus orbitae* [17]. In the lama, the *proc. zygomaticus ossis temporalis* also does not reach the *margo orbitalis*. It is described that the *proc. temporalis ossis zygomatici* is bifurcated, enclosing the *proc. zygomaticus ossis temporalis* above and below [18].

The *lamina perpendicularis ossis palatini*, described thus far only in carnivores [17], clearly contributes to the medial wall of the *orbita* in the alpaca. In other domestic species, it is generally described as forming part of the medial wall of the *fossa pterygopalatina*, located ventral to the *orbita*, with its dorsal portion extending dorsally to contribute to the boundary of the *orbita* [22].

The *facies orbitalis maxillae* is present in the alpaca and has likewise been described in the cat and horse [23, 24].

The *foramina* identified in the alpaca are largely consistent with those described in other mammals. The *for. ethmoidale* is singular in alpacas and lamas [18, 19], whereas two *foramina* are typically present per side in carnivores [24].

The *can. opticus* is present in the alpaca and has been described in various domestic mammals [17, 23] as well as in lamas [18].

Similarly, the *for. orbitorotundum* is present in ruminants and pigs, whereas horses possess separate *fissura orbitalis* and *for. rotundum* [17]. The presence of a *for. orbitorotundum* in the alpaca therefore corresponds to the condition described in ruminants and has likewise been reported in the lama [18], indicating a shared morphological pattern of the medial wall of the *orbita*.

### 4.2 About the *Mm. bulbi*

The *mm. bulbi* facilitate coordinated three‐dimensional rotation of the bulbus oculi within the *orbita*, enabling precise ocular positioning [16, 25].

The composition of these muscles of the alpaca, consisting of the four *mm. recti*, the *m. levator palpebrae superioris*, the two *mm. obliqui*, and the *m. retractor bulbi* has been described in this form on several occasions, for example, in horses and cattle [16, 26] as well as in dromedaries [27].

The origin, course, and insertion of the four *mm. recti* of the alpaca correspond to those of domestic mammals, as does the *m. levator palpebrae superioris* [26].

The *m. retractor bulbi* also originates and runs as described in the existing literature [28]. Although some studies describe the *m. retractor bulbi* of domestic animals as being more or less distinctly divided into four parts [29], other reports indicate a greater degree of species‐specific variation in its subdivision. In cattle, the muscle has been described as incompletely divided [7], whereas in carnivores, the *m. retractor bulbi* consists of four distinct portions [30, 31, 32]. In the alpaca, the *m. retractor bulbi* forms a uniform muscular cone, with the only interruption occurring at the passage of the *n. opticus*.

The origin and course of the two *mm. obliqui* in the alpaca correspond to the descriptions found in domestic mammals [26, 33].

An interesting anatomical variation compared to the descriptions of the *mm. bulbi* of carnivores, horses and ruminants is seen in the bowhead whale, in which the muscle fibers, except for the *m. retractor bulbi*, insert not only into the *bulbus oculi* but also radiate into the *palpebrae*, presumably to expand the field of vision through additional eyelid movement [34].

### 4.3 On the *Nn. craniales* in the *Orbita*

The course of the *n. opticus* of the alpaca, including its entry into the muscle cone between the *m. rectus dorsalis* and *m. rectus medialis*, has also been described in the cat [31]. As in other domestic mammals, it then enters the *m. retractor bulbi* and reaches the *bulbus oculi* under the protection of this muscle [26].

The division of the *n. oculomotorius* into a *r. dorsalis* and a stronger *r. ventralis* is likewise consistent in the alpaca. However, the literature describes the *r. dorsalis*, in addition to the *n. abducens*, as contributing a branch to the *m. retractor bulbi*, which differs from our findings: In this study, it is the *r. ventralis* that provides a branch to this muscle [26].

The innervation of the *m. levator palpebrae superioris* proved difficult to trace, given its origin at the *m. rectus dorsalis*. A similar description is given for the cat [31].

A study in human anatomy confirmed that the *m. levator palpebrae superioris* as well as the *m. rectus superior* are involved in coordinated movement during vertical saccades due to their shared innervation, with the *m. rectus superior* also contributing to the elevation of the *palpebra superior* [35]. Based on the observed innervation pattern, it may be hypothesized that the *m. rectus dorsalis* contributes to the elevation of the *palpebra superior* in the alpaca. This assumption is supported by the close association between the *m. rectus dorsalis* and the *m. levator palpebrae superioris*, as branches supplying the latter were observed to course through the former. The similar innervation patterns of both muscles may indicate a degree of functional coordination during eyelid elevation.

The *n. trochlearis* innervates the *m. obliquus dorsalis* not only in the alpaca, but also in the dromedary [36] and domestic mammals such as the dog, cat, horse, cattle, and small ruminants [26, 37].

The dichotomy of the *n. trochlearis* observed in some specimens in this study has not been previously reported in the anatomical literature for these mammals [26, 37].

However, a more recent study in humans, rhesus monkeys, rabbits, and cattle demonstrated that all species examined exhibited a bifurcation of the *n. trochlearis* just prior to its entry into the *m. obliquus superior*. Each branch innervates a distinct, nonoverlapping compartment of the muscle fibers, enabling selective control over vertical or torsional eye movements [38]. The observed bifurcation of the *n. trochlearis* in the alpaca may indicate the presence of a comparable compartmental innervation pattern of the *m. obliquus dorsalis*, although further studies are required to confirm this hypothesis.

The *n. ophthalmicus* and its three main branches—*n. frontalis*, *n. lacrimalis*, and *n. nasociliaris*—can be traced in the alpaca in a manner comparable to that described in domestic mammals [26].

The *r. communicans cum n. lacrimali* of the *n. zygomaticus* observed in the alpaca has not been described in the dog, but the other domestic animals [26].

A particularly interesting finding is the delicate connection between the *n. ophthalmicus* and the *r. ventralis n. oculomotorii* in the alpaca. Similarly, the fine branch of the *n. ophthalmicus* directed to the *m. rectus dorsalis* is not reported in this form in domestic mammals [26].

The innervation pattern of the *n. abducens* in the alpaca corresponds to that of domestic mammals. However, the penetration of the *m. rectus lateralis* by this nerve, as observed in the alpaca, has not been described in this way in other species [37]. A different innervating pattern has been described in the dromedary, where the *n. abducens* is not only innervating the *m. rectus lateralis* and the *m. retractor bulbi* but also the *m. rectus dorsalis* [27].

### 4.4 Limitations and Outlook

The length and diameter of the *mm. bulbi* and *nn. craniales* were not measured in this study. As the specimens were initially stored in brine, potential tissue swelling could not be excluded. Future studies should obtain morphometric measurements prior to brine storage to ensure accuracy.

Histological examination was not possible, as no tissue samples were collected. The carcasses arrived at the institute with a postmortem delay, so the integrity of histological structures could no longer be guaranteed. Future investigations should include histological sampling, particularly in the region of the “fontanela lagrimal“, to evaluate the presence of potential preorbital glands.

Histological analysis of the fine branches of the *n. ophthalmicus* could further clarify their functional significance and precise topographical relationship to the *mm. bulbi*. Likewise, examination of the bifurcation of the *n. trochlearis* could determine whether the alpaca exhibits compartmental innervation of the *m. obliquus dorsalis*.

Further studies should also evaluate the clinical applicability of the present anatomical findings. Cadaveric injection studies using contrast‐enhanced imaging could be used to identify optimal approaches for orbital nerve blocks, including the Peterson nerve block, and to assess the distribution of injectates within the orbit. Similar techniques have been applied in bovine cadavers to compare orbital nerve block techniques [8] and may contribute to the refinement of regional anesthesia in alpacas while improving procedural accuracy and reducing the risk of iatrogenic injury.

## 5 Conclusion

Despite the growing importance of alpacas as domestic and farm animals, anatomical research on their orbital region remains limited, which can undermine clinicians' confidence in diagnosis and surgical procedures. To the best of our knowledge, this study provides the first comprehensive macroscopic description of the alpaca's bony *orbita*, *mm. bulbi*, and *nn. craniales*, directly addressing this increasingly critical knowledge gap.

Notably, we identified several anatomical nuances that distinguish the innervation of the alpaca *orbita* from that of other domestic mammals. The *n. trochlearis* bifurcates shortly before entering the *m. obliquus dorsalis*, a feature not commonly reported in other domestic mammals. Additionally, fine branches of the *n. ophthalmicus* were observed to connect with the *m. rectus dorsalis*. One branch divided into two filaments: one appeared to approach the *r. ventralis* of the *n. oculomotorius* but continued to the m. rectus ventralis, while the other filament coursed toward the *m. retractor bulbi*. Contrary to previous literature, describing innervation of the *m. retractor bulbi* via the *n. abducens* and the *r. dorsalis* of the *n. oculomotorius*, our findings indicate that the *n. abducens* and the *r. ventralis* of the *n. oculomotorius* contribute to its innervation.

Apart from these minor differences, we demonstrated a high degree of similarity in the orbital structures to other domestic animals. This comparability allows veterinarians to apply established anatomical frameworks to the alpaca and provides a solid foundation for both further research and clinical practice—ultimately enhancing confidence and precision in clinical decision‐making.

## Author Contributions

**Sophia Weis:** conceptualization, investigation, writing – original draft, writing – review and editing, visualization, methodology, validation. **Johann Maierl:** supervision, resources, validation.

## Funding

The authors have nothing to report.

## Ethics Statement

Only postmortem carcass donations from private owners or veterinary clinics were utilized for this study, and therefore, approval from an ethics committee was not required. All owners agreed to donate their pet's bodies after death and to their use for scientific purposes.

## Conflicts of Interest

The authors declare no conflicts of interest.

## Data Availability

The macroscopic images analyzed during the current study are available from the corresponding author upon reasonable request.
